# Stem cell models in ataxia-telangiectasia

**DOI:** 10.4103/NRR.NRR-D-25-00988

**Published:** 2025-12-30

**Authors:** Maria Talmon, Giulia Lecchi, Luigia G. Fresu

**Affiliations:** 1 Department of Pharmaceutical Sciences, University of Piemonte Orientale, Novara, Italy; 2 Department of Health Sciences, School of Medicine, University of Piemonte Orientale, Novara, Italy

**Keywords:** ataxia-telangiectasia, ataxia-telangiectasia mutated, genetic disease, *in vitro* cell modeling, neurodegenerative disorders, neurons, rare disease, skeletal muscle cell, stem cells, urine-derived stem cell

## Abstract

Ataxia-telangiectasia is a rare neurodegenerative disease with a complex phenotype, which has recently been associated with alterations in metabolism, inadequate responses to oxidative stress and inflammation, as well as increased cardiovascular and tumor risk. All of these appear to be attributable to genetic mutations/variants in the ataxia-telangiectasia mutated gene, which encodes the ataxia-telangiectasia mutated protein. The possibility of a better phenotypic definition provides a basis for timely, personalized therapeutic intervention to reduce or prevent worsening of clinical symptoms. Several ataxia-telangiectasia mutated knock-out murine models were created, but none efficiently developed progressive ataxia, failing to recapitulate human neurodegeneration following ataxia-telangiectasia mutated deficiency. Furthermore, considering the strong awareness of the ban on the use of animals in scientific research, a great effort has been made and is still ongoing to create human cellular models of ataxia-telangiectasia with the aim of understanding in detail the molecular mechanisms of neurodegeneration and skeletal muscle defect, of being able to identify specific therapies. This review highlights human stem cell approaches as *in vitro* models that have been established as attempts to study the outcomes of ataxia-telangiectasia mutated inactivation regarding neurogenic and myogenic differentiation. The first attempts at differentiation from fetal tissues, through the induced pluripotent stem cell revolution and the latest urine-derived stem cells will be reviewed.

## Introduction

**Figure 1:**
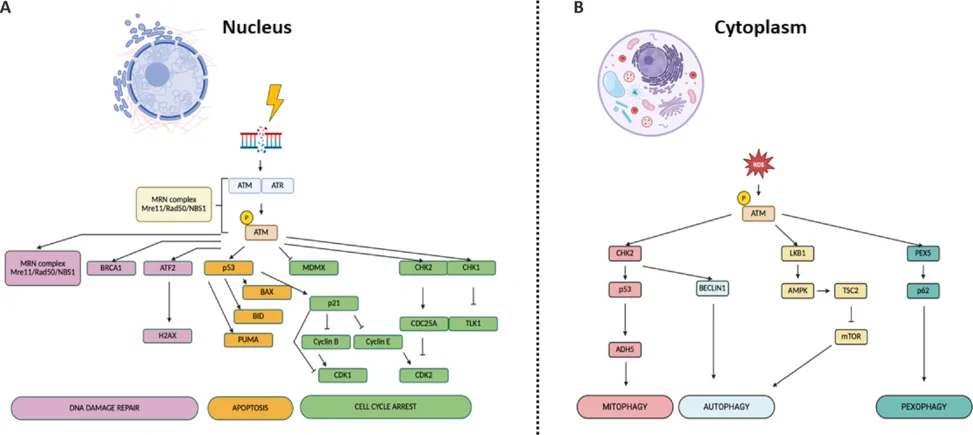
Roles of ATM in cellular repair and mitochondrial oxidative stress. (A, B) Role of ATM in the nucleus (A) and cytoplasm (B). Created with BioRender.com. ADH5: Alcohol dehydrogenase 5 (class III); AMPK: AMP-activated protein kinase; ATF2: activating transcription factor 2; ATM: ataxia telangiectasia mutated; ATR: ataxia telangiectasia and Rad3-related; BAX: BCL2-associated X protein; BECLIN1: coiled-coil myosin-like BCL2-interacting protein; BID: BH3 interacting domain death agonist; BRCA1: breast cancer gene 1; CDC25A: cell division cycle 25A; CDK1: cyclin-dependent kinase 1; CDK2: cyclin-dependent kinase 2; CHK1: checkpoint kinase 1; CHK2: checkpoint kinase 2; H2AX: H2A histone family member 2; LKB1: serine/threonine kinase 11; MDMX (also known AS MDM4): MDM4 regulator of P53; MRE11: meiotic recombination 11; mTOR: mammalian target of rapamycin; NBS1 (also known as NBN): nibrin; p21: cyclin dependent kinase inhibitor 1A; p53: tumor protein p53; p62: sequestosome 1; PEX5: peroxisomal biogenesis factor 5; PUMA: p53 upregulated modulator of apoptosis/BCL2 binding component 3; TLK1: tousled like kinase 1: TSC2: tuberous sclerosis 2 protein.

Ataxia-telangiectasia (A-T) is one of the relatively most frequent forms of infantile ataxia caused by mutations/variants of the ataxia-telangiectasia mutated (*ATM*) gene [located on chromosome 11q22.3], which determines either the deficiency of the ATM protein or its kinase activity (Savitsky et al., 1995). The *ATM* gene encodes the ATM protein, which is a member of the phosphatidyl-inositol-3-kinase family, enzymes that respond to DNA damage by phosphorylating substrates involved in DNA repair mechanisms and cell cycle control (Awasthi et al., 2015; Paull, 2015; Lee and Paull, 2021). A-T typically presents with ataxia, with clinical onset in the first months of life, and cerebellar degeneration, which becomes anatomically evident later (Tavani et al., 2003; Lai et al., 2024). Other manifestations include premature aging of tissues, leading to hyperpigmented spots on the skin (Dong et al., 2022; Haj et al., 2025), ocular telangiectasias (Gattermeyer-Kell et al., 2025), and an increased incidence of childhood and adult-onset tumors, even in pediatric patients (Viart et al., 2025). Immunodeficiency (Giovannetti et al., 2002; Driessen et al., 2013; Desimio et al., 2021) is common, resulting in increased susceptibility to pulmonary infections and increased sensitivity to ionizing radiation. Furthermore, cardiovascular dysplasia, cirrhosis-like liver disease, nutritional deficiencies, and a higher frequency of immune-mediated diseases, such as diabetes and thyroiditis, may occur (Rothblum-Oviatt et al., 2016). All the clinical signs are associated with the role of ATM in DNA damage repair mechanisms and cell cycle regulation, and also to the multifaceted functions of the ATM signaling pathway, involving several intracellular proteins and cascades in both the nucleus and cytoplasm (Dong et al., 2022), as detailed in **Figure 1A** and **B**, respectively. In the nucleus, ATM is mainly involved in DNA repair response with cell cycle arrest, and/or apoptosis. Following DNA damage from various origins, ATM phosphorylates p53, and through the degradation of MDMX and in collaboration with CHK2, arrests the cell cycle. Subsequently, ATM collaborates with the MRN complex (Mre11/Rad50/NBS1) to increase the expression of BRCA1 and AFT2, which are necessary for DNA repair. If the damage is too severe to be repaired, ATM also directly phosphorylates p53 to induce apoptosis and eliminate the defective cell. In the cytoplasm, ATM acts primarily as a sensor of oxidative stress, can promote autophagy through ATM/CHK2/BECLIN1 and ATM/LKB1/AMPK/TSC2/mTOR pathway, mitophagy with ATM/CHK2/p53/ADH5 cascade, and pexophagy with ATM/PEX5/p62 signaling pathway.

In the absence of ATM or in the presence of a mutated protein that is not fully functional, the failure to control the cellular redox state leads to an accumulation of reactive oxygen species (ROS) and impaired mitochondrial function, resulting in ATM-deficient cells eventually dying. This appears to be the mechanism underlying most of the clinical signs in A-T, among which cerebellar and hippocampal neurodegeneration that compromises the activity of skeletal muscle, which is under direct control of the central nervous system, with a progressive decline in motor activity, leading to immobility and the use of wheelchairs in young A-T patients (Miterko et al., 2021). The mechanism by which ATM controls the redox state of cells can occur in 2 ways: cytosolic ATM induced by an increase in ROS has been shown to translocate to the nucleus where it inhibits the expression of components of the mitochondrial redox complex (Zhang et al., 2018); but evidence has shown that a part of ATM localizes directly in the mitochondria and regulates homeostasis by controlling the levels of ROS mediating antioxidant effects (Leeson et al., 2024). The vital involvement of microglia in cerebellar dysfunction has been demonstrated, both in murine and human cell models. Lai et al. (2024) demonstrated microglial inflammation in the cerebellum of A-T patients, responsible for the release of neurotoxic cytokines.

Although functional alterations in skeletal muscle are considered secondary to neuronal damage, Dematteis et al. (2025) have demonstrated a direct role of ATM in skeletal muscle cells, as fully detailed below.

Despite the extensive knowledge about the role of ATM in various cellular processes, the exact mechanism leading to the onset of A-T is not yet fully defined. Therefore, more work is needed to integrate all aspects and gain a more comprehensive understanding of the pathophysiology of A-T by identifying appropriate experimental models. In particular, in line with the recent position of the National Institution of Health (NIH) to move away from animal models (https://www.nih.gov/news-events/news-releases/nih-prioritize-human-based-research-technologies), the search for new experimental strategies in human cell–based technologies such as organoids, tissue chips, and computational models, represents a priority not only from an ethical point of view but also to improve the accuracy in drug development studies to ensure safer therapies and in the study of pathological conditions hard to translate from animal to humans. The A-T falls in these situations. Indeed, A-T mouse models recapitulate neuronal abnormalities but not the neurodegeneration or the telangiectasia (Sacks et al., 2018). For example, Tassinari et al. (2019) studied the direct role of ATM absence in skeletal muscle defects using an ATM-knockout (KO) mouse model and observed reduced muscle mass and fiber size, increased muscle atrophy, and enhanced mitochondrial ROS production, but this model did not reproduce neurodegeneration and central nervous system defects, likely due to early mortality of animals. Therefore, alternative *in vivo* models have been developed to overcome some of these limitations, but always with little success. Interestingly, the porcine model generated by Beraldi et al. (2015) exhibited a delay in growth, Purkinje cell loss, and alteration in the cytoarchitecture. Conversely, rat models have mimicked neurodegeneration features associated with A-T, including motor neuron loss and progressive paralysis (Quek et al., 2017). However, no animal model has fully replicated the complete human A-T phenotype. Overall, *in vivo* models contributed to deepening our knowledge of the pathophysiology of A-T, but ethical considerations and interspecies limitations that often complicate data interpretation need to be addressed (Cendelin et al., 2022; Domínguez-Oliva et al., 2023).

Therefore, only by integrating the results obtained by various animal models could we fully understand the complexity of A-T, but this would require significant resource consumption. In this scenario, new human cell models represent an added value. This review describes the evolution of the human stem cell approaches, from fetal tissues, through the induced pluripotent stem cell (iPSC) revolution and the latest urine-derived stem cells, established as an attempt to study the outcome of ATM inactivation in relation to the neuronal and skeletal muscle differentiation, and to further our understanding of the molecular mechanisms of neurodegeneration and skeletal muscle defect in A-T.

## Data Sources

The articles cited in this narrative review were obtained through a computer-based online search of the Web of Science and PubMed databases. The initial article screening was performed by combining the following keywords: “ataxia-telangiectasia,” “ataxia telangiectasia mutated protein,” “ATM,” *“in vitro* cell model,” “animal models,” “stem cells,” “urine-derived stem cells.” The selected articles focused on *in vitro* and *in vivo* study models of ataxia telangiectasia, highlighting the potential of stem cells as a human *ex vivo* model. Some articles were deemed irrelevant based on the analysis of the title and abstract and therefore excluded.

## Stem Cell Model for Ataxia-Telangiectasia Mechanistic Studies

**Figure 2:**
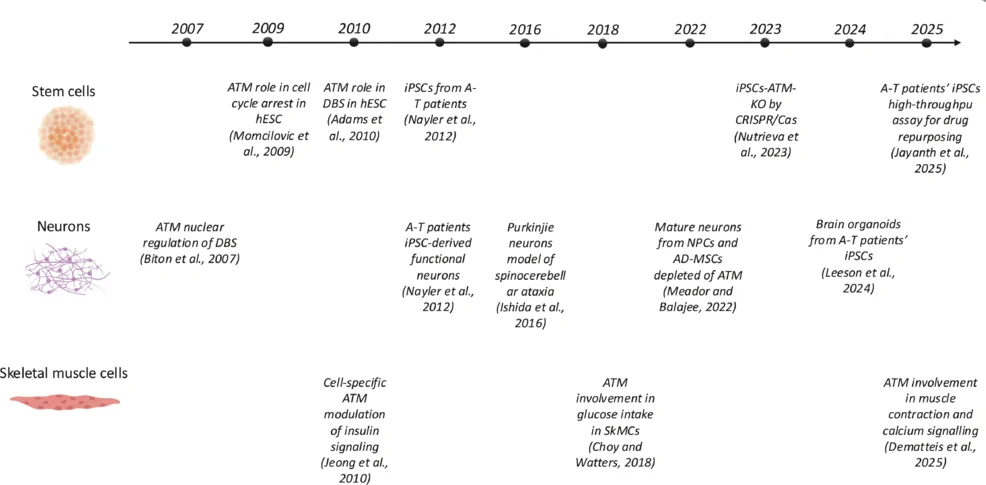
Progress and use of stem cells in A-T modeling. The figure represents a timeline of key milestones in stem cell, neuronal, and skeletal muscle model evolution for the A-T study, as described in the review. Created with BioRender.com. AD-MSCs: Adipose mesenchymal stem cells; A-T: ataxia-telangiectasia; ATM: ataxia-telangiectasia mutated; CRISPR: clustered regularly interspaced short palindromic repeats; DSB: double-stand break; hESC: human embryonic stem cell; iPSCs: induced pluripotent stem cells; KO: knockout; NPCs: neural progenitor cells; SkMCs: skeletal muscle cells.

The wide range of clinical manifestations of A-T requires a deeper analysis of the tissue-specific role of ATM and downstream cascades, both to gain new insights into the pathology and to identify new potential drug targets. Therefore, it is essential to have human cell models that are easily isolated, maintained, and bioengineered. Among the various cell models under study, stem cells represent an excellent solution as they can be used to study the expression and the role of ATM in undifferentiated cells and during differentiation processes, while terminally differentiated cells, such as neurons and muscle cells, are efficient in recapitulating tissue-specific defects consequent to the ATM deficiency. **Figure 2** exhibits the timeline of key milestones of the development of stem cell models for A-T described in this review.

In the first decade of 2000, the role of ATM in cell cycle arrest and repair of DNA double-stand breaks (DSBs) has been well described in human ESCs. Momčilović et al. (2009) demonstrated that in Υ-irradiated human embryonic stem cells (hESCs), ATM properly localizes near the DSB, thus promoting the G2 cell cycle arrest (Momcilović et al., 2009). Conversely, when ATM was chemically inhibited, the G2 cell cycle arrest was completely compromised (Momcilović et al., 2009). Thus, this model highlighted the role of ATM in early stem cells, although DSB repair is predominantly orchestrated by the ATR kinase rather than ATM in hESCs (Adams et al., 2010). However, it is important to underline that ESCs, for their function, are characterized by a more sophisticated replication regulation mechanism than in other stem cells to protect genomic stability in view of the development of a healthy embryo (Blanpain et al., 2011); indeed, not only does ATM regulates the ATR-dependent pathway, however, as demonstrated by Blakemore et al. (2021), ATM itself is under the control of Mybl2, which in hESCs is in fact expressed up to a thousand times more, thus suggesting that in an ATM-KO hESC model, pathogenetic characterization may be more complicated. More recently, the analysis of the impact of ATM deficiency on stem cells has moved from hESCs to iPSCs, which offer the significant advantage of being potentially derived from A-T patients, thus allowing for a patient- and mutation-specific cell model. Currently, several iPSC lines genetically modified (Meador and Balajee, 2022; Nurieva et al., 2023; Zhang et al., 2023) or generated from A-T patients (Nayler et al., 2012; Leeson et al., 2021, 2024; Jayanth et al., 2025) are available in a cell repository (**Table 1**).

**Table 1:** List of deposited iPSC cell lines

Cell line	Cell source	Gene/Locus	Genotype/Mutation	Repository	Reference
AIBNi014-A	Human olfactory neurosphere derived cells	*ATM* gene/11q22.3	Exon 10 homozygous splice acceptor variant: C>CT (NM_000051.3:c.1236-3dupT; RCV000159602.2) Exon 44 heterozygous frameshift (insertion) variant: C>CTT (NM_000051.3:p.Arg2136fs/c.6404_6405insTT; RCV000131770.2)	https://hpscreg.eu/cell-line/AIBNi014-A	Leeson et al., 2021
SCVI2655 (SCVIi083-A) - iPSC	PBMCs	*ATM* gene/11q22.3	SCVIi083-A: c.4143dup	https://hpscreg.eu/cell-line/SCVIi083-A	Zhang et al., 2023
SCVI2591 (SCVIi084-A) - iPSC	PBMCs	*ATM* gene/11q22.3	SCVIi084-A: c.5697C > A	https://hpscreg.eu/cell-line/SCVIi084-A	Zhang et al., 2023
Cell line 1: PEIi019-A-13	Fibroblasts	*ATM* gene/11q22.3	Homozygous point mutation C->T in exon 3 at R35 in the ATM gene. Introduction of R35* stop codon	https://hpscreg.eu/cell-line/PEIi019-A-13	Nurieva et al., 2023
Cell line 2: PEIi019-A-14	Fibroblasts	*ATM* gene/11q22.3	Homozygous point mutation A->C in exon 63 at K3016 in the ATM gene. Introduction of K3016Q	https://hpscreg.eu/cell-line/PEIi019-A-14	Nurieva et al., 2023
Cell line 3: PEIi019-A-15	Fibroblasts	*ATM* gene/11q22.3	Homozygous point mutation T->C in exon 29 at L1465 in the ATM gene. Introduction of L1465P	https://hpscreg.eu/cell-line/PEIi019-A-15	Nurieva et al., 2023
Cell line 4: PEIi019-A-16	Fibroblasts	*ATM* gene/11q22.3	Homozygous point mutation C->T in exon 63 at A3054 in the ATM gene. Introduction of A3054V	https://hpscreg.eu/cell-line/PEIi019-A-16	Nurieva et al., 2023

ATM: Ataxia-telangiectasia mutated; iPSC: induced pluripotent stem cell; PBMCs: peripheral blood mononuclear cells.

On the Human Pluripotent Stem Cell Registry (hPSCreg; https://hpscreg.eu/), several engineered iPSC cell lines are registered, each carrying an ATM mutation reported in individual A-T patients (Leeson et al., 2021; Nurieva et al., 2023; Zhang et al., 2023). iPSCs have been generated both from healthy donors and then gene edited, and from A-T patients, for example: fibroblasts from healthy donors have been reprogrammed to generate iPSCs, then modified by CRISPR/Cas9 technology to obtain a panel of missense and nonsense ATM mutations (Nurieva et al., 2023). In particular, the modified iPSCs exhibited biallelic point mutations introducing a stop codon (cell line: PEIi019-A-13) or a change in aminoacidic sequence (K3016Q, cell line: PEIi019-A-14; L1465, cell line: PEIi019-A-15; A3054, cell line: PEIi019-A-16) of the ATM protein; and iPSCs obtained from breast cancer patients carrying ATM mutations (ClinVar 181880 and 421488 (Zhang et al., 2023). iPSCs from A-T patients could also be procured from the National Institute of Neurological Disorders and Stroke (NINDS) Human Cell and Data Repository, where two pluripotent cell lines from A-T fibroblasts carrying various point mutations are available (https://stemcells.nindsgenetics.org/) (Jayanth et al., 2025).

Other than the cell line register, several research groups took advantage of iPSCs to gain new insights into A-T. Leeson et al. (2021) generated pluripotent cells from olfactory biopsies of A-T patients describing two novel mutations in ATM gene leading to a truncated non-functional protein: (i) heterozygous frameshift variants resulting from a TT insertion in exon 44 (ClinVar: RCV000131770.2) and (ii) homozygous splice acceptor variants by T insertion at the splice acceptor site prior to exon 10 (ClinVar: RCV000159602.2). Although a reduced reprogramming efficiency, all the ATM mutated iPSCs exhibited good properties of stemness and self-renewal, representing a valuable platform for studying the A-T molecular basis (Nayler et al., 2012; Zhang et al., 2023), mirroring the ATM deficiency phenotype. Moreover, the A-T iPSCs exhibited an altered radiation-induced signaling and radiosensitivity. Following γ-irradiation, DNA synthesis in A-T iPSCs was inhibited by 20%, while DNA repair in the control group was inhibited by 60%. This indicated that DNA damage repair signaling was impaired in the absence of ATM. Additionally, A-T iPSCs exhibited cell cycle checkpoints defects and alterations in genes related to mitochondrial and pentose phosphate pathways (Nayler et al., 2012, 2017). iPSCs derived from A-T patients have also been used to create a high-throughput drug repurposing assay, which measures phosphorylated CHK2 as a reliable marker of the DNA damage response (Jayanth et al., 2025). This assay screened approximately 6000 compounds, identifying 8 potential hits for further investigation. These hits might lead to the development of new therapeutic strategies to alleviate A-T symptoms (Jayanth et al., 2025).

In recent years, a few attempts have been made to generate A-T models starting from adult stem cells. In 2022, Meador and Balajee established two models, starting from human neural stem/progenitor cells (ReNcell, Temecula, CA, USA) and adipose mesenchymal stem cells (Invitrogen, San Diego, CA, USA). Neural stem/progenitor cells were transduced by a lentivirus carrying shRNA for ATM to largely abolish the protein synthesis, while adipose mesenchymal stem cells were treated with an ATM-specific inhibitor (KU-55933) to minimize ATM kinase activity (Meador and Balajee, 2022). Once irradiated, these models suggest that the ATM kinase is required for the optimal efficiency of DSB repair and viability in human stem cells and its role is exacerbated in neuronal cells. These models have been instrumental to better highlight the role of ATM in progenitor cells, which present a phenotype more susceptible to oxidative stress and prone to accumulating harmful mutations. Another interesting adult stem cell type used as a platform for A-T mechanistic studies is urine-derived stem cells (USCs) (Dematteis et al., 2025). USCs were isolated from healthy donors and gene-edited using CRISPR/Cas9 technology to obtain the KO of the ATM protein, creating a model for ataxia-telangiectasia. These mutated USCs efficiently recapitulate the known defects of the disease, and this model has provided new insight into molecular alterations (Dematteis et al., 2025). Specifically, USC-ATM-KO cells exhibited cell cycle alterations both at the basal status and after UV exposure, as well as a decrease in late apoptosis, indicating an inability to stop growing and repair a DNA insult (Dematteis et al., 2025). Moreover, ATM depletion increased mitochondrial oxidative stress. This model allowed for the assessment of the role of ATM in calcium homeostasis, as USC-ATM-KO cells displayed impaired mitochondrial calcium uptake, resulting in a calcium overload in the cytosol (Dematteis et al., 2025). A key advantage of USCs, compared to other adult stem cells, is their easy and painless collection. This simple collection method allows researchers to obtain multiple samples from A-T patients, even repeatedly and in close succession, without any dangerous side effects or risks to the donors, regardless of age or disease stage. Furthermore, while USCs have a limited capacity for cell division, they offer a straightforward way to create patient-specific cell models. This method avoids genetic modification and long culture times, unlike the process for creating iPSCs. To study a genetic disease in a mutation-specific manner, minimal genetic manipulation could represent a consistent advantage.

In summary, for both iPSCs and adult stem cells, two main strategies have been pursued to generate reliable A-T models: (i) isolation of cells directly from A-T patients; (ii) gene editing of healthy donor cells or cell lines, and both have advantages and disadvantages. Patient-specific models enable a personalized investigation of the disease and, in the future, could be instrumental in establishing a personalized therapy. However, the A-T models derived from patients lack an appropriate isogenic unaffected control, making it challenging to distinguish disease-specific phenotypes from underlying genetic variability. In several works, cells isolated from a healthy family member were used as an unaffected control to patient iPSCs (Itzhaki et al., 2011; Nayler et al., 2012), but this did not consider epigenetic and genetic differences between individuals. In this article, the gene editing technologies can generate mutated cells and, contemporarily, refer to the un-edited cells as a control. This approach certainly highlights the effects of ATM mutations while avoiding genetic background interference, but ATM mutations can lead to various outcomes: null, truncated, or kinase-dead proteins with different cellular consequences (Jenni et al., 2024). Therefore, although generating a null model is relatively straightforward, creating cell lines representing the different mutations of a disease as complex and heterogeneous as A-T could require extensive and time-consuming work.

All together, these works highlight adult stem cells as a valuable tool for recapitulating genetic defects and uncovering novel pathogenetic mechanisms, which may represent a novel therapeutic target in the future.

## Stem Cell Model for Neuronal Study

Neurological symptoms are the main clinical manifestations of A-T, and it is therefore necessary to have reliable models that recapitulate the central nervous system phenotype of the ataxia. A number of human neuronal cellular platform have been developed and several strategies were explored starting from both A-T patient stem cells (Carlessi et al., 2013b; Leeson et al., 2024; Jayanth et al., 2025) and healthy cells in which ATM was chemically inhibited (Biton et al., 2007; Carlessi et al., 2013b; Meador and Balajee, 2022), silenced by shRNAs (Biton et al., 2007; Carlessi et al., 2013b; Meador and Balajee, 2022), or knocked out by gene editing technology (Yeo et al., 2021). These varied methods provide a comprehensive view of the effects of molecular changes in A-T. They enable the study of the location of ATM in both the nucleus and cytoplasm, creating models that reflect both the kinase and the trophic functions of the protein. To analyze the correlation between ATM subcellular localization and its role in DSB response, Biton et al. (2007) generated neuronal-like cells starting from hESCs and neural stem cells isolated from fetal cerebral cortex. From both stem cell lines, mature neurons have been obtained expressing most of the ATM into the nucleus, where it acts as a master regulator of DSB response through the activation of phosphorylation cascade (Biton et al., 2007). Both the chemical inhibition of ATM by the KU-55933 inhibitor and the knockdown by shRNA negatively affect the DNA repair regulation (Biton et al., 2007), demonstrating that this process is strictly dependent on the ATM kinase activity. To further investigate the role of ATM in DNA damage induced by ionizing radiation, human mature neurons have been generated starting from a neural progenitor cell line silenced for ATM and adipose MSCs treated by ATM-specific inhibitor through the induction of neurosphere formation (Meador and Balajee, 2022). In mature neurons, ATM kinase resulted to be essential to resolve the ionizing radiation-induced DNA damage. In fact, a reduced expression of genes involved in base excision and DSB repair was observed in both models. Moreover, the kinetic activity of ATM was demonstrated to be fundamental to maintain the genome integrity during the neurogenesis process, interplaying with other PI-3 related kinases, ATR, and DNA-PK (Meador and Balajee, 2022). Neuronal cells and brain organoids have also been established starting from olfactory mucosa-derived iPSCs of A-T patients (Leeson et al., 2024). In particular, the organoids have been generated by the induction of spheroid formation, embedded in Matrigel, differentiated and cultured for about 100 days. This 3D system has efficiently modeled A-T defects as the transcriptomic analysis on organoids revealed a downregulation of the genes correlated to the DNA repair, in line with a non-functional ATM protein (Leeson et al., 2024). Moreover, the KEGG pathway analysis pinpointed strong mitochondrial impairments in mutant brain organoids, including upregulation of oxidative phosphorylation and Kreb cycle pathways, as well as increases in mRNAs coding for genes involved in ROS metabolism, neurodegeneration, and cellular senescence (Leeson et al., 2024). Mitochondrial defects shed the light on ATM cytoplasmic localization near the mitochondria, supporting its role in the organelle homeostasis regulation (Yeo et al., 2021). This is due to the ATM involvement in the assembly of IP3R1-GRP75-VDAC1 complex, leading to a defective tethering between ER and mitochondria (Yeo et al., 2021). To better contextualize whether the ATM involvement in ER-mitochondria interaction has a relevant role in A-T neurodegeneration, further studies on cerebellar neurons need to be performed.

Another key aspect in A-T neuronal model development is the possibility of setting specific protocols to obtain specific neurons. Thus, it will be possible to dissect the role of ATM in different areas of the brain both in neurogenesis and cell maintenance. Indeed, since ATM deficiency does not appear to interfere with *in vitro* neuronal differentiation, it could influence the phenotype of established neurons. Carlessi et al. (2013a) differentiated iPSCs from fibroblasts of A-T patients into neural progenitor cells and then generated both neuronal and glial progeny. They demonstrated that starting from ATM-defective neural progenitor cells, a comparable number of MAP2 and β-tubulin III expressing neurons has been obtained, while the GABAergic phenotype is strongly attenuated as demonstrated by a reduction in the amount of Υ-aminobutyric acid positive cells. This observation strongly reflects the GABA signaling impairment in the cerebellum of A-T patients (Perry et al., 1984). A limitation of this study and of the current available *in vitro* A-T neuronal models is the lack of a well-defined protocol to obtain Purkinje neurons, the cell type most affected by ATM deficiency in A-T. Some attempts have been pursued to model spinocerebellar ataxia in which progressive Purkinje neuron depletion and cerebellar deterioration are the main clinical manifestations, but due to the complexity of cultivating these cells, further research efforts are necessary to establish a reproducible and effective protocol (Ishida et al., 2016; Kamei et al., 2023; Naor et al., 2024).

## *In Vitro* Stem Cell Model for Skeletal Muscle Study

Muscle atrophy and choreoathetosis usually appear in the first decade of the life of A-T patients; therefore, understanding the pathological mechanisms underlying these alterations is fundamental (Tassinari et al., 2019). Although researchers have widely studied the neurological aspects of A-T (Leeson et al., 2024), the skeletal muscle component is still not very explored despite some evidences have highlighted the role of ATM in muscle biology, in particular in mitochondrial homeostasis, in cellular differentiation and in response to oxidative stress (Ching et al., 2013; Blignaut et al., 2019; Dematteis et al., 2025), and its absence has vital consequences. Choy and Watters (2018) demonstrated that an ATM deficiency in skeletal muscle cells is associated with a reduced translocation of Glut4 to the cell membrane after insulin stimulation, followed by severe energy defects given the high need to intake glucose in muscle cells. Additionally, they have developed a cellular model in which the deficiency of ATM leads to a defect of mitochondrial homeostasis, with an increase in the level of ROS production, and consequent compromise of the mitophagy and apoptosis in skeletal muscle cells (Choy and Watters, 2018). Moreover, ATM has recently been implicated in the stress-induced Ca^2+^ signaling, implying a direct impact of ATM on the muscle contraction (Yeo et al., 2021). This evidence demonstrates the involvement of ATM in the development of skeletal muscle diseases. Therefore, it is important to further investigate the effects of ATM absence. Immortalized myogenic cell lines and iPSCs have been particularly delved into (Nayler et al., 2012; Tassinari et al., 2019). The former (e.g., murine C2C12 and rat L6 myotubes), obtained by either the KO of the ATM gene or derived from ATM-KO mice, offer an effective platform for the study of muscle pathology, and they have been employed to demonstrate that ATM-deficient muscle was characterized by an impairment in the insulin signal transduction pathway (Jeong et al., 2010). Interestingly, they observed differential roles of ATM in these two cell lines. For example, ATM was shown to modulate insulin-induced Akt phosphorylation in C2C12 murine cells, but not in L6 myotubes, suggesting a cell-type-specific function (Jeong et al., 2010). Additionally, other cell lines have been used; for instance, a dual lentiviral transduction (employing both an hTERT insert and a tet-inducible MYOD insert) was used to immortalize fibroblasts from both wild-type and A-T patients. This experimental approach has made it possible to obtain more homogenous cell lines that allow researchers to perform repetitive experiments and long-term analyses to study muscular dystrophies. However, up to now, no muscle cell lines obtained by transdifferentiation of fibroblasts have been employed to investigate A-T disease (Almeida et al., 2021).

Building on these considerations, iPSCs derived from patients with A-T have been demonstrated to be a promising approach. A-T-iPSCs have been reprogrammed from somatic cells, thereby providing the original genomic alterations of the patient. A previous study corroborated that A-T-iPSCs retain A-T-features, such as radiation sensitivity and genomic instability (Fukawatase et al., 2014). It is interesting to note that Ovchinnikov et al. (2020) used the CRISPR/CAS9 technology to restore the ATM functionality in iPSC derived from fibroblasts of A-T patients, with the correction of DNA damage response, the reduction of mitochondrial reactive species production, and sensitivity to oxidative stress.

These promising findings suggest that gene-corrected A-T-iPSCs could serve as a helpful tool for differentiation analyses and functional comparisons with their uncorrected counterparts, as demonstrated by the study of Nayler et al. (2017), in which A-T-iPSCs have been successfully differentiated in neuronal lineages, marking their relevance for the study of the neurodegenerative aspects of A-T. Nonetheless, the potential of A-T-iPSCs to differentiate into skeletal muscle cells remains largely unexplored (Ovchinnikov et al., 2020). Additionally, although the pluripotency of iPSCs allows the differentiation to various types of cells, thus supporting the concept of modeling of the disease and therapeutic development, obtaining iPSCs in general requires invasive procedures (for example, blood samples and skin biopsies), which can be demanding given the early onset of A-T (Fukawatase et al., 2014).

However, the myogenic potential remains a gap in the A-T study area, especially considering that iPSC-derived skeletal muscle cells have been previously employed in modeling other neuromuscular disorders, such as Duchenne muscular dystrophy and limb-girdle muscular dystrophies (Al Tanoury et al., 2021; Bruge et al., 2022; Delafenêtre et al., 2024). Overall, immortalized myogenic cell lines and iPSCs should not be seen as opposing methods, but rather as complementary tools for modeling rare genetic diseases. Immortalized lines provide a stable, easy-to-handle, and reproducible platform to perform long-term functional assays, whereas iPSCs hold the advantage of recapitulating patient-specific genetic backgrounds and being able to differentiate into multiple cell types. Taken together, these models widen the number of platforms available to researchers and allow for a more detailed evaluation of the pathological mechanisms underlying A-T and other rare disorders.

Furthermore, USCs, whose characteristics and advantages have been described previously (Xu et al., 2019; Talmon et al., 2022; Cavaleiro et al., 2023; Sun et al., 2024), have proven to be a successful tool to study skeletal muscle defects. Dematteis et al. (2025) have established an A-T cellular model by generating an ATM-KO in USCs isolated from healthy donors, using the CRISPR/Cas9 technology and differentiated into skeletal muscle cells via a MyoD-inducible lentiviral vector. A vital observation was that this cellular skeletal muscle model displayed increased muscle contractility. Indeed, by stimulating with acetylcholine, an altered increase in contractility has been demonstrated in ATM-deficient skeletal muscle cells when compared to the wild-type counterpart. This phenomenon aligned with a higher ATP-induced Ca^2+^ release in the cytoplasmic compartment of ATM-KO cells, paralleled by a lower Ca^2+^ intake into the mitochondria (Dematteis et al., 2025). These results involve a muscle-intrinsic component of the ATM protein, which could thus demonstrate what the consequences of its absence in muscles in a pathology such as A-T may be and emphasize the potential of USC-based models for therapeutic analyses. While these *in vitro* systems mimic vital A-T cellular aspects, more investigation is required to fully recapitulate the skeletal muscle background *in vivo*. Anyway, USCs isolated from A-T patients, carrying individual mutations, will open new opportunities for the preclinical testing of pharmacological or gene therapy approaches to restore muscle function in A-T.

## Conclusions

Cell modeling represents a new era for studying human pathologies, especially thanks to the advancement in stem cell technologies. Stem cells can be recovered from various patient samples, typically through invasive procedures, and then reprogrammed or differentiated into specific cell types. This allows researchers to study physio-pathological mechanisms or to search for new treatments. CRISPR/Cas9 genome engineering in stem cells has further expanded the scope of this cellular model, particularly for studying rare genetic diseases, such as A-T.

Human stem cell models have proven fundamental to discerning complex A-T features and certainly A-T-iPSCs are a promising platform to dissect ATM-related to neuron and muscle dysfunction. Nevertheless, the non-invasive accessibility and the capacity of USCs to reproduce key muscular phenotypes serve as a significant advancement in A-T research. Future research should focus on refining these cellular platforms for translational research applications, including investigating ATM-related neuron dysfunction. This could bridge a critical gap in existing disease models and ultimately provide novel therapeutic strategies for this rare disease.

## Data Availability

*Not applicable.*
